# A Comprehensive and Comparative Study of *Wolfiporia extensa* Cultivation Regions by Fourier Transform Infrared Spectroscopy and Ultra-Fast Liquid Chromatography

**DOI:** 10.1371/journal.pone.0168998

**Published:** 2016-12-30

**Authors:** Yan Li, Ji Zhang, Tao Li, Honggao Liu, Yuanzhong Wang

**Affiliations:** 1 Institute of Medicinal Plants, Yunnan Academy of Agricultural Sciences, Kunming, Yunnan, China; 2 Yunnan Technical Center for Quality of Chinese Materia Medica, Kunming, Yunnan, China; 3 College of Resources and Environment, Yuxi Normal University, Yuxi, Yunnan, China; 4 College of Agronomy and Biotechnology, Yunnan Agricultural University, Kunming, Yunnan, China; National University of Ireland - Galway, IRELAND

## Abstract

Nowadays, *Wolfiporia extensa* as a popular raw material in food and medicine industry has received increasing interests. Due to supply shortage, this species of edible and medicinal mushroom has been cultivated in some provinces of China. In the present study, cultivated *W*. *extensa* collected from six regions in Yunnan Province of China were analyzed by an integrated method based on Fourier transform infrared (FT-IR) spectroscopy and ultra-fast liquid chromatography (UFLC) coupled with multivariate analysis including partial least squares discriminant analysis (PLS-DA) and hierarchical cluster analysis (HCA) in order to investigate the differences and similarities in different origins and parts. In the tested mushroom samples, characteristic FT-IR spectra were obtained for acquiring comprehensive fuzz chemical information and pachymic acid was determinated as a biomarker in the meantime. From the results, the comparison of samples was achieved successfully according to their geographical regions and different parts. All the samples displayed regional dependence and the inner parts showed better quality consistency. In addition, the chemical constituents of cultivated *W*. *extensa* could be also affected by the cultivation methods. Meanwhile, there was an interesting finding that the soil properties of cultivation regions may have a relationship with the chemical constituents of the epidermis of soil-cultured *W*. *extensa*, rather than the inner parts. Collectively, it demonstrated that the present study could provide comprehensive chemical evidence for the critical complement of quality evaluation on the cultivated *W*. *extensa*. Moreover, it may be available for the further researches of complicated mushrooms in practice.

## Introduction

Edible mushrooms have been regarded as important resources of delicacies and nutraceuticals across the globe since they are quite rich in carbohydrates, digestible protein, essential amino acids, vitamins and minerals with some having unique flavors and aromas [1, 2]. These mushrooms are either collected from the wild fields e.g. *Cantharellus cibarius*, *Boletus edulis*, *Inonotus obliquus* or cultivated artificially such as *Agaricus bisporus*, *Lentinula edodes* and *Agaricus subrufescens* etc. [3]. A number of these species have appeared as mushrooms products on the shelves in supermarkets (S1 Fig) and been cooked in the daily life (S2 Fig). In recent years, artificial cultivation of edible mushrooms with the benefits of short growing times, very low inputs requirements for production, prevailing external climatic and easy production technologies has become more and more popular in many regions due to an increasing awareness on the usage of them [4–6]. The quality of cultivated edible mushrooms is becoming an object of public concern.

However, studies have shown that the special status of edible mushroom products is directly associated with the complexity of entire chemical components and different varieties of bioactivities of the raw materials, which are somewhat jagged according to locations, species and cultivation methods, even cultivated areas with the disparity of environmental conditions such as agricultural soil and climate [7–11]. In the previous study, Chen et al. [11] analyzed the cultured fruiting bodies of *Ganoderma lucidum* and proved that the chemical properties of *G*. *lucidum* from the same collection site were much similar to each other and all the samples could be differentiated based on the origins, suggesting the quality of *G*. *lucidum* was relative with the location. As reported by Jing et al. [12], the quality of polysaccharides in the fruiting bodies of *Flammulina velutipes* from three provinces in China were assessed and significant differences could be found in the tested samples. Similarly, some researchers also reported that the *Ophicordyceps sinensis* obtained from different geographical origins could be discriminated by the levels of mannitol, trehalose and some amino acids [13]. Therefore, the places from which the edible mushrooms collected are crucial if the specimens are to be of good qualities.

Until now, in order to investigate the differences and elucidate the quality of edible mushrooms with different geographical areas, many scientists are devoted to developing suitable qualitative and quantitative techniques for the chemical components in edible mushrooms, including spectroscopic and chromatographic methods which are focused on the characterization of a complex system of a tested sample [14]. With the technological advances, recent studies move towards the trend of the combination between spectroscopic and chromatographic methods for better analysis of biological samples in diverse fields including food, plants, mushrooms, drugs etc. with the aid of appropriate multivariate analyses [14–16]. For example, Pan et al. [17] developed FT-IR combined with UFLC-MS/MS to profile raw and processed *Gentiana rigescens*, suggesting this method could provide an effective platform for monitoring quality variations of *G*. *rigescens* under different processed approaches. According to Liu et al. [18], a comprehensive strategy based on MIR, UV and chromatographic technique was carried out to monitor the quality consistency of Weibizhi tablet. For the mushrooms, some researchers reported that IR spectroscopic and HPLC chromatographic fingerprints in combination with chemometrics could be used to determine the quality of *F*. *velutipes* fruiting bodies [12]. The spectroscopic approaches are rapid, simple and cost-effective to analyze the holistic chemical components based on structural information about the compounds [19, 20]. But it is merely one side of the coin. This method has some limitation such as it fails to directly determinate the variation on major constituents and their contents. In some cases, monitoring the characteristic constituents is also important to sustain more specific properties of samples [21]. Fortunately, such a possibility is offered by the chromatographic methods which could provide detailed quantitative information about the chemical composition of the samples as well as provide complementary information which can lead to a better understanding of spectral signatures [22, 23]. The integration of these two approaches is more comprehensive and detailed, and has shown strong potential to investigate the differences and assess the quality of edible mushrooms with different geographical origins.

*Wolfiporia extensa*, called “*fu-ling*” in Chinese, is a well-known edible and medicinal mushroom widely used in China and other East Asian countries [24]. This species has been not only used in traditional Chinese prescriptions with some other herbs [25], but also popularly regarded as a sub-material for the traditional food called “*fu-ling jiabing*” in Beijing, China (S3 Fig). Phytochemical and pharmacological studies have revealed that the major chemical components of *W*. *extensa* were triterpenoids and polysaccharides, especially the pachymic acid which belonged to the triterpenoid compounds has received more attentions because of the various biological activities including anti-emetic, anti-inflammatory properties as well as potential anti-cancer [26–28]. As an important mushroom resource, *W*. *extensa* is widely distributed in many provinces of China. It is worth mentioning that Yunnan Province of China is recognized as one of the main genuine regions of this species. Due to the gradually increasing market demand and supply shortage, nowadays *W*. *extensa* of different cultivation origins and methods have flooded into the market. In this context, investigating the differentiation and assessing the quality of *W*. *extensa* with different cultivation origins is of great economic importance, especially for completely manufactured in given geographical regions following traditional methods, which may add characteristics and commercial values to the mushroom products, making it more competitive in the market.

In the present study, we qualitatively and quantitatively investigate the differences and similarities in different parts of cultivated *W*. *extensa* originated from six regions in Yunnan Province of China using a tandem technique based on FT-IR combined with UFLC. Characteristic FT-IR spectra were obtained and pachymic acid was determinated as a biomarker in the tested mushrooms. The entire joint data were subjected to multivariate analysis including supervised PLS-DA and unsupervised HCA for the integrity comparison among all the samples. The results could provide and broaden the knowledge on current studies on the quality evaluation of cultivated *W*. *extensa*.

## Materials and Methods

### Sample Collection and Initial Preparation

**Fig 1:**
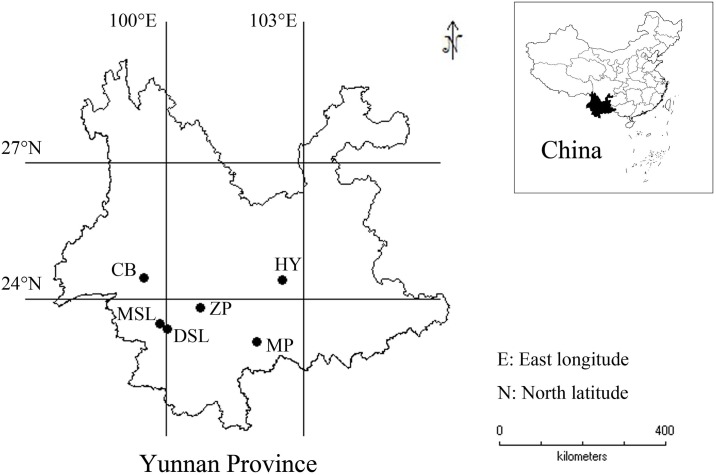
The sampling location of cultivated *W*. *extensa* in Yunnan Province.

The fresh sclerotia of *W*. *extensa* samples were obtained from six regions in Yunnan Province of China during the collecting seasons recorded in Chinese Pharmacopoeia (version 2015) [29] (Fig 1, Table 1). All of them were cultivated species. In particular, samples from Hongta District in Yuxi were cultivated via soil-less pattern, growing on the sawn-off roots of old, dead pine trees covered by plastic films in an indoor environment (S4 Fig). Whereas, other mushroom samples were covered by red or yellow soils in the course of the cultivation in the fields. All the samples were authenticated by Dr. Honggao Liu (College of Agronomy and Biotechnology, Yunnan Agricultural University, Kunming, China) and the voucher specimens were preserved in the specimen room of Institute of Medicinal Plants, Yunnan Academy of Agricultural Sciences. No specific permits were required for the described field studies, as no endangered or protected species were sampled, and the localities where the samples came from are not protected in any way.

**Table 1:** Information of the *W*. *extensa* samples.

No.	Code	Collection site	Latitude	Longitude	Elevation (m)	Description	Soil color of collection site
1–5	HY	Hongta District, Yuxi	N24°25'54.7"	E102°31'5.6"	1720	Inner part	—
6–10	CB	Changning County, Baoshan	N24°28'8.5"	E99°30'11.4"	2011	Inner part	Yellow
11–15	ZP	Zhenyuan County, Pu’er	N23°49'12"	E100°44'26.8"	1892	Inner part	Red
16–20	DSL	Dawen Township, Shuangjiang County, Lincang	N23°20'55.5"	E100°0'17.1"	1438	Inner part	Yellow
21–25	MSL	Mengmeng Town, Shuangjiang County, Lincang	N23°28'40.5"	E99°50'16.1"	1052	Inner part	Yellow
26–30	MP	Mojiang County, Pu’er	N23°4'3.5"	E101°58'35.5"	1979	Inner part	Red
31–35	HY	Hongta District, Yuxi	N24°25'54.7"	E102°31'5.6"	1720	Epidermis	—
36–40	CB	Changning County, Baoshan	N24°28'8.5"	E99°30'11.4"	2011	Epidermis	Yellow
41–45	ZP	Zhenyuan County, Pu’er	N23°49'12"	E100°44'26.8"	1892	Epidermis	Red
46–50	DSL	Dawen Township, Shuangjiang County, Lincang	N23°20'55.5"	E100°0'17.1"	1438	Epidermis	Yellow
51–55	MSL	Mengmeng Town, Shuangjiang County, Lincang	N23°28'40.5"	E99°50'16.1"	1052	Epidermis	Yellow
56–60	MP	Mojiang County, Pu’er	N23°4'3.5"	E101°58'35.5"	1979	Epidermis	Red

Initially, all the fresh sclerotia samples were cleaned by a soft brush and air-dried in the shade for approximately two months, which is the traditional method in China for thousands of years [30]. Then, they were further dried in an oven at 50°C for 12 hours to a constant weight. After that, each of them was separated into two parts—the inner part (“*bai fu-ling*” in Chinese) and the epidermis (“*fu-ling pi*” in Chinese). Each sample was pulverized in a porcelain mortar and screened using a 100-mesh stainless steel sieve. The sieved powders were stored in new sealed polyethylene bags and kept in a dry and clean condition until further analysis.

### FT-IR Spectroscopy and Spectral Data Processing

For FT-IR spectral analysis, each sample powder was mixed uniformly with the spectroscopic grade potassium bromide (KBr) powder, in which the ratio of sample to KBr was 1.5:100 (w/w), and then milled and pressed to form a tablet for testing. The infrared spectra were recorded from 4000 to 400 cm^-1^ with 16 scans per spectrum at 4 cm^-1^ resolution using the Frontier FT-IR spectrometer (Perkin Elmer, USA) equipped with a deuterated triglycine sulfate (DTGS) detector. In the meantime, the interferences of the absorbance bands of carbon dioxide and water in the atmosphere were deducted by ratioing the background spectra which collected using pure dried KBr in tablet form. Each sample was recorded in triplicate and the averaged spectrum was used for next analysis. Then, the digitalized original averaged FT-IR spectra were pre-processed, including automatic baseline correction and spectral intensity normalization by using Omnic 8.0 software (Thermo Nicolet, USA). Automatic baseline correction could automatically correct the tilted baselines of the selected spectra with the baseline points selected by the software in order to minimize the problem caused by baseline drift as well as increase the accuracy of spectra simultaneously. The spectral intensity normalization could firstly shift the spectrum vertically to bring the lowest Y value to 0 absorbance, regardless of whether the lowest value was currently above or below 0. The software then multiplied the spectrum by a scaling factor to make the highest value 1 absorbance unit. In the experiment, a thick sample of a material absorbs more infrared energy than a thin sample, resulting in greater peak heights. Normalizing the spectra of the samples compensates for this pathlength effect so that we can compare their peak heights. After that, Savitzky-Golay (SG) filter and first derivative were used to maximize existing differences of FT-IR spectra. In the case of SG filter, the number of data points was 15. For the first derivative, the number of data points was also 15 and the polynomial order was quadratic.

### UFLC Analysis

The content of pachymic acid in *W*. *extensa* was determined base on the verified method described in our previous study [31]. Briefly, dried sample powder (0.5 g) was accurately weighed before putting into a 10.0 mL test tube and 5.0 mL of methanol was added to the tube. Following ultrasound-assisted extraction at 30°C for 1 h, the extracted solution was stored at 4°Cand filtrated through a 0.22 μm membrane filter (Millipore, USA) prior to UFLC analysis. For the establishment of calibration curve of pachymic acid, the stock solution of this analyte (1000 μg/mL) was prepared in methanol individually and then diluted with methanol to appropriate concentrations (77.60, 130, 216, 600 and 1000 μg/mL) for the next analyses.

The analyses of methanol extracts were performed using a Shimadzu LCMS-8030 ultra-fast liquid chromatography system (Shimadzu, Kyoto, Japan) equipped with a UV detector, an autosampler, a triple quadrupole mass spectrometer detector via an electrospray ionization (ESI) interface and binary gradient pumps. System control and data analysis were processed with Shimadzu LabSolution software (Shimadzu, Japan). The chromatographic separation was carried out on a Shim-pack XR-ODS III column (1.6 μm, 75 × 2.0 mm) using 0.1% formic acid in water (A) and acetonitrile (B) as mobile phase at a flow rate of 0.45 mL/min. Sixty *W*. *extensa* samples were analyzed under the application of the following gradient program: 0–2.15 min, 46% B; 2.15–2.65 min, 46–55% B; 2.65–7.00 min, 55–60% B; 7.00–7.85 min, 60–78% B; 7.85–9.75 min, 78–95% B; and 9.75–12.00 min, remaining at 95% B. The temperature of the column oven was set at 45°C and the injection volume of each sample was 2.0 μL as well as the detective wavelength of pachymic acid was selected at 210 nm. Each methanol extract of tested sample was determined upon three replicate injections and the averaged content of pachymic acid in each *W*. *extensa* sample was used for the further analysis.

### Statistical Analysis

The data of contents of pachymic acid in *W*. *extensa* samples were analyzed using GraphPad Prism software (ver. 6.0, GraphPad Prism Inc., San Diego, CA, USA). The comparisons of the contents of pachymic acid among the same part samples with different origins were analyzed by analysis of variance (ANOVA) while the contents of standard in the two different parts of samples from the same collection site were compared by *t*-test. The level of significance defined as 95% (P < 0.05). What’s more, the quantitative determination and full FT-IR spectra data were integrated and subjected to multivariate analysis including supervised PLS-DA and unsupervised HCA which were performed by software named SIMCA-P^+^ 13.0 (Umetrics, Umeå, Sweden) in order to evaluate the relationship in terms of similarity or dissimilarity among different samples.

## Results and Discussion

### Chemical Assignments of Absorption Bands in FT-IR Spectra of Cultivated *W*. *extensa*

**Fig 2:**
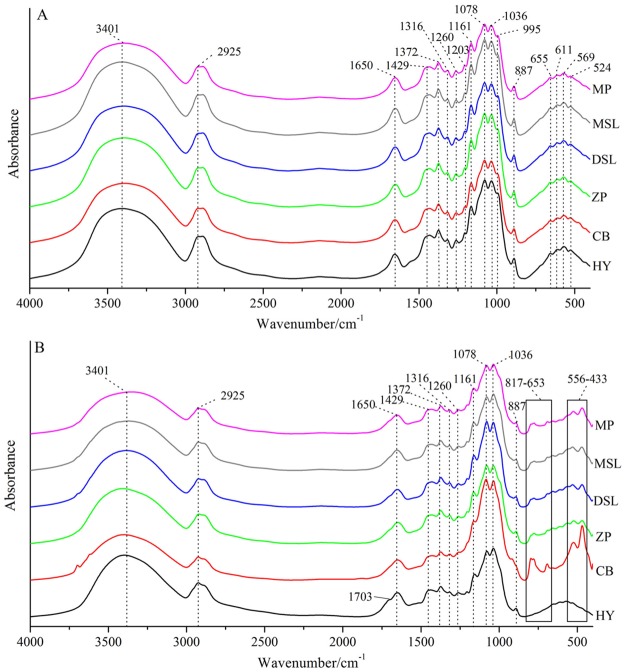
Averaged original FT-IR spectra of the inner part (A) and epidermis (B) of cultivated *W*. *extensa* samples from each collection site.

Averaged raw FT-IR spectroscopic spectra for the different parts of cultivated *W*. *extensa* samples from each collection site are presented in Fig 2. A number of observations of several characteristics may be made on these spectra, which are typically considered for the initial comparison of samples. As can be seen from the spectra of inner part samples (Fig 2A), similar spectral characteristics are observed with a total of 17 major common peaks. Starting from the higher wavenumber, the broad band at 3401 cm^-1^ is mainly attributed to the stretching vibration of O-H of triterpene, polysaccharide and sterol. The common backbone of C-H and -CH_2_ vibration can be seen at 2925 cm^-1^, which is revealed in all of the spectra. Amide I is a dominant band in the region of 1700–1600 cm^-1^, arising from the C-N stretching and C = O stretching vibrations at 1650 cm^-1^ while the amide III band is present at 1260 cm^-1^, which suggest the estimation of protein components [32]. Furthermore, the lower part of FT-IR spectra is mainly interpreted as the carbohydrate. The peak at 1372 cm^-1^ is definitely assigned as triterpene compounds (CH_2_ = CH-CH_3_) and peaks in the region of 1200–900 cm^-1^ appears to be identified as C-O-H bending band, -CH_2_ bending band, C-C stretching band and C-O stretching band, which are mainly attributed to the deformation of carbohydrate [33, 34]. What’s more, the C-C stretching around at 1078 and 1036 cm^-1^ probably indicate the structures in chitin, a major structural polysaccharide in mushrooms [35]. For the region of 900–400 cm^-1^, it is mainly assigned to the existence of polysaccharides, such as *β*-D-glucan, the pyranose form of glucose and so on [36–38]. In addition, the characteristic peak at around 887 cm^-1^ is probably attributed to the C = CH_2_ presented in triterpenes [39]. Overall, no significant spectral differences among the inner parts are observed, suggesting the existence of similar chemical components in these samples.

From the examination of the spectral profiles recorded for the epidermis samples (Fig 2B), not only the similar spectral features have been presented, but the differences in some particular bands are visible. The region from 4000 to 850 cm^-1^ contains 11 obvious common peaks whereas several differences appear at 850–400 cm^-1^, particularly in the regions of 817–653 cm^-1^ and 556–433 cm^-1^, which are also the main distinguishing spectral characteristics between some epidermis and inner part samples. What’s more, samples from Hongta District in Yuxi have shown the most distinct differentiation because the averaged spectrum of these samples has the obvious characteristic peak at 1703 cm^-1^ which appears to be assigned to the existence of -COOH in triterpenes [40–42] and it also has few peaks in the 850–400 cm^-1^ region. It is possible that the peak at 1703 cm^-1^ also exists in the other spectra but may be overlapped. Besides, samples with the strongest intensity in the regions of 817–653 cm^-1^ and 556–433 cm^-1^ are obtained from Changning County of Baoshan while the spectra of other samples reveal the similarity of each other in these regions. It indicated that geographical origins were likely to cause the changes in the chemical components of epidermis. Consequently, the chemical constituents of cultivated *W*. *extensa* could be affected by different parts and the collection regions, which is similar to the previous studies of edible mushrooms [43, 44]. Moreover, the geographical origins may have different influences on the chemical component of inner part and epidermis of *W*. *extensa*.

### Quantitative Determination of Pachymic Acid in Cultivated *W*. *extensa*

The developed UFLC method was subsequently applied to the quantitation of pachymic acid in different cultivated *W*. *extensa* samples. The calibration curve (*y* = 1500.8348 *x*– 11750.6286) which constructed by plotting the peak area against concentration was in the range of 77.60–1000 μg/mL with good coefficient of determination (*r*^2^ = 0.9998). The limit of detection (LOD, S/N = 3) and quantification (LOQ, S/N = 10) were 20.41 and 68.02 μg/mL, respectively.

**Fig 3:**
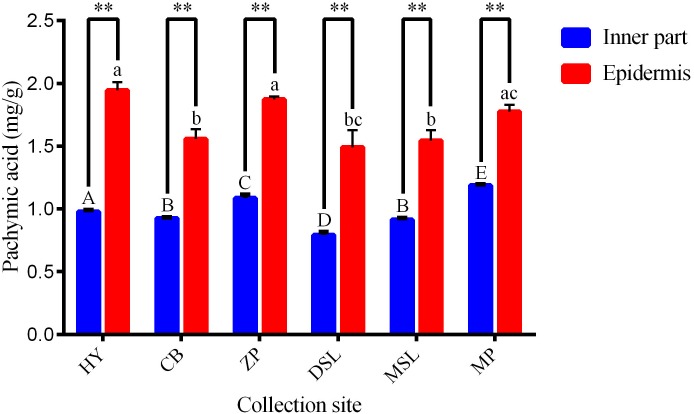
Averaged contents (mg/g) of pachymic acid in *W*. *extensa* with different regions (n = 5). Different superscripts on the bar with the same color indicate significant differences among the same part samples (*P* < 0.05). **, the comparison between two different parts of *W*. *extensa* collected from the same site based on *t*-test (*P* < 0.01).

Samples obtained from six different regions were analyzed and the averaged contents of pachymic acid in samples of each collection site are summarized in Fig 3. According to the results, samples sourced from Mojiang County of Pu’er have shown the highest averaged content of pachymic acid (1.19 ± 0.01 mg/g) in all the inner part samples, followed by samples from Zhenyuan County of Pu’er (1.08 ± 0.02 mg/g) and samples with the lowest content were collected from Dawen Township, Shuangjiang County of Lincang (0.79 ± 0.02 mg/g). What’s more, significant differences (*P* < 0.05) are presented clearly among the inner part samples with different collection sites except that from Changning County of Baoshan and Mengmeng Town, Shuangjiang County of Lincang. Comparatively, for the epidermis, the contents of the investigated analyte in samples from Hongta District of Yuxi as well as Zhenyuan and Mojiang Counties of Pu’er are similar and that of other samples have no statistically significant differences. Overall, the contents of pachymic acid of all the inner part samples (range from 0.79 to 1.19 mg/g) are lower than that of the epidermis (range from 1.49 to 1.94 mg/g), which also shows statistically significant differences (P < 0.01) between the two parts of *W*. *extensa* collected from the same site based on *t*-test. In addition, it still demonstrates that the difference in the content of pachymic acid between the two part samples depends on the collection regions. Obviously, the least differentiation between the two parts is 0.59 mg/g which presented in the samples with the collection area of Mojiang County of Pu’er whereas the most one is shown in the samples from the region of Hongta District in Yuxi with the value of 0.97 mg/g. More interesting, both the inner part and the epidermis *W*. *extensa* collected from Dawen Township, Shuangjiang County of Lincang display the lowest content of the analyte in the same part samples. In a word, the contents of pachymic acid in samples may change with the geographic origins and different parts, which may be relative to their qualities.

### Partial Least Squares Discriminant Analysis

In order to visualize the differences among tested samples, it is of importance to extract and rationalize information from any multivariate description of them. In this study, one of the supervised pattern recognition techniques, named PLS-DA was performed as a preliminary step for carrying out a detailed investigation among a pool of samples, which could maximize the separation among pre-defined classes as well as select the variables considered to be influential for classification [45]. The parameters R^2^Y and Q^2^ were used to evaluate the efficiency of this method and it suggested good potential for predicting class membership when the value of Q^2^ was greater than 0.5 [46]. The data of content of pachymic acid and FT-IR spectra were integrated by orthogonal signal correction in order to remove the interference spectral information which was unrelated with the pachymic acid. Afterwards, the entire joint data were imported into the software for PLS-DA analysis.

**Fig 4:**
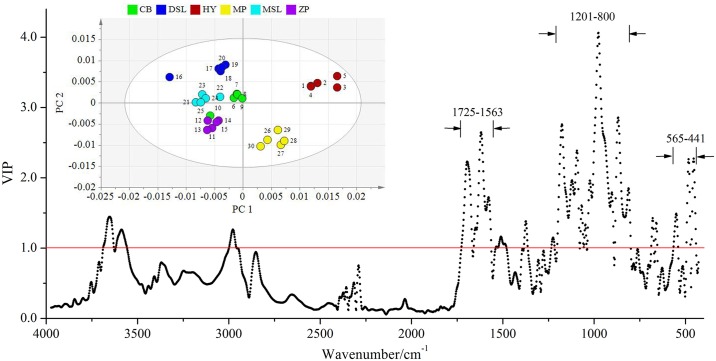
VIP plot with an inserted PC 1-PC 2 score plot showing the separation of the inner part samples.

For the result of inner part samples, the first seven principal components (PCs) of PLS-DA were the optimal choice with the R^2^Y and Q^2^ parameters calculated to be 89.3% and 74.6%, respectively. As shown in Fig 4, the inserted two-dimension score plot with 95% confidence ellipses separates the inner part *W*. *extensa* into six groups. Obviously, PC 1 is determined mainly by positive scores for the samples obtained from Hongta District of Yuxi and Mojiang County of Pu’er, especially the former ones are predominantly far from all the others. Individual samples belonging to the same geographical origin are more closely grouped in discrete clusters, indicating that PLS-DA is able to discern between collection sites and the inner part samples may have regional dependence. From the corresponding misclassification table (Table 2), the classification accuracy of six *W*. *extensa* is all 100%. In addition, to gain more insight into the differences among samples, the variable importance in the projection (VIP) values were examined. The variables exhibiting a VIP score above a value of 1.0 given by PLS-DA were selected because they were considered to be significantly contributive to the discrimination of geographical origins [47]. As is evident in Fig 4, after data fusion, the variables that play the greatest role in discriminating inner part samples mainly distribute in the regions of 1725–1563, 1201–800 and 565–441 cm^-1^, which comprise approximately 68% of the total 475 variables. According to the previous studies, the spectral region from 1725 to 1563 cm^-1^ mainly indicated the structures of triterpenes and a few protein components [32, 48] while the other two regions may suggest the presence of polysaccharides [49]. Thus, it could be presumably inferred that the differentiation of the inner part samples may be primarily related to the diversity of triterpenes and polysaccharides.

**Table 2:** Misclassification table of the inner part samples using the developed PLS-DA method.

	Members	Correct	HY	CB	ZP	DSL	MSL	MP	No class (YPred < 0)
HY	5	100%	5	0	0	0	0	0	0
CB	5	100%	0	5	0	0	0	0	0
ZP	5	100%	0	0	5	0	0	0	0
DSL	5	100%	0	0	0	5	0	0	0
MSL	5	100%	0	0	0	0	5	0	0
MP	5	100%	0	0	0	0	0	5	0
No class	0		0	0	0	0	0	0	0
Total	30	100%	5	5	5	5	5	5	0

**Fig 5:**
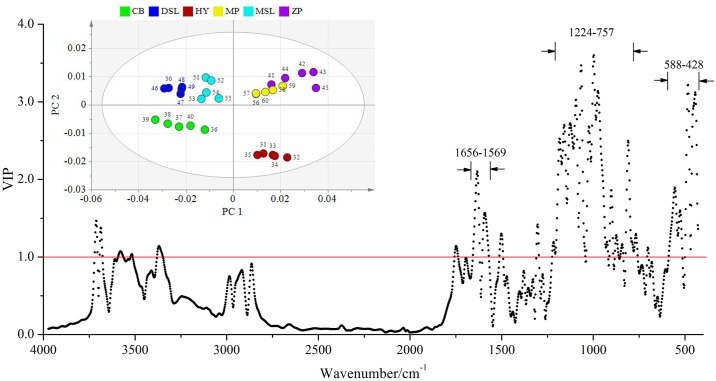
VIP plot with an inserted PC 1-PC 2 score plot containing the separation of the epidermis samples.

Thereafter, PLS-DA was also used to evaluate the relationships in terms of similarity of dissimilarity among the epidermis samples. The values of R^2^Y and Q^2^ were 76% and 70.3%, respectively with the first five PCs, which also showed good predictive capacity of this method. As can be seen from Fig 5, the score plot shows good clustering, and PC 1 and PC 2 are determined as good criteria for discriminating the epidermis samples with different geographical origins. All the samples are well distributed into five classes in the two-dimensional space. Similarly, the strong positive scores on PC 1 are found for samples which obtained from Hongta District of Yuxi. In addition, samples with the collection sites of Zhenyuan and Mojiang Counties of Pu’er are also distributed on the right-hand side of the plot with strong positive scores on PC 1. In contrast, other samples are separated on the left-hand side of the plot, with negative scores in this PC. In the misclassification table (Table 3), the classification accuracy rate of all the samples is found to be 100%, suggesting PLS-DA could show a good separation among the epidermis samples of *W*. *extensa* from different origins. Likewise, the regional dependence may occur in these samples. What’s more, from the VIP plot (Fig 5), variables in three regions (1656–1569, 1224–757 and 588–428 cm^-1^) are responsible for the separation, and particularly 70% of these variables are included in the last two regions which may show the presence of polysaccharides according to the literature [50]. Hence, it could be presumably concluded that the differences of the epidermis samples may be mainly related to the polysaccharides.

**Table 3:** Misclassification table of the epidermis samples using the developed PLS-DA method.

	Members	Correct	HY	CB	ZP	DSL	MSL	MP	No class (YPred < 0)
HY	5	100%	5	0	0	0	0	0	0
CB	5	100%	0	5	0	0	0	0	0
ZP	5	100%	0	0	5	0	0	0	0
DSL	5	100%	0	0	0	5	0	0	0
MSL	5	100%	0	0	0	0	5	0	0
MP	5	100%	0	0	0	0	0	5	0
No class	0		0	0	0	0	0	0	0
Total	30	100%	5	5	5	5	5	5	0

**Fig 6:**
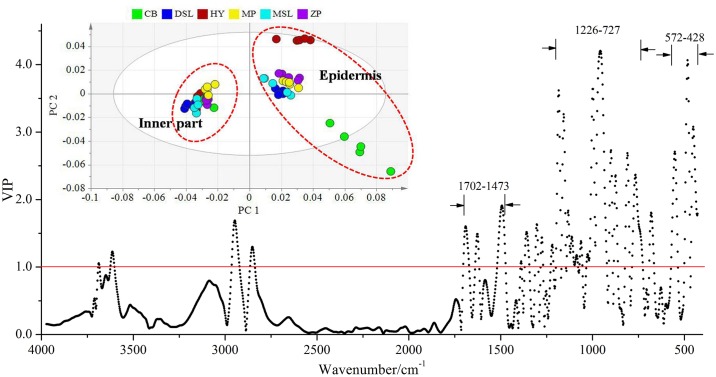
VIP plot with an inserted PC 1-PC 2 score plot of different parts of *W*. *extensa*.

Generally, as recorded in Chinese Pharmacopoeia (versions 2015), the inner part and epidermis of *W*. *extensa* had different efficacies and they should be differently used in practice [29]. This principle was confirmed based on the chemical approach in our study. PLS-DA also showed a good predictive capacity with the R^2^Y and Q^2^ calculated to be 95.9% and 95.2% (first four PCs), respectively. Observing Fig 6, the score plot from PLS-DA separates all the investigated *W*. *extensa* samples into two major classes. One class consisted of the inner part samples whereas the other one consisted of the epidermis, suggesting a clear differentiation between these two parts. Both of the classification accuracy rates are 100% (Table 4). Furthermore, most of the epidermis samples are distributed relatively in accordance with their collection sites. On the contrary, it is worth noting that despite collected from different origins, the inner part samples are clustered more closely, which may be due to the similarity of their chemical constituents and also represent the quality consistency of these samples. In addition, it could be inferred that the two part of *W*. *extensa* revealed different regional dependence and quality consistency. Briefly, the epidermis showed better regional dependence and comparatively the inner part displayed better consistency in quality. In the VIP plot (Fig 6), the variables in the wavenumber ranges of 1702–1473, 1226–727 and 572–428 cm^-1^ which may be in agreement with the presence of polysaccharides and few triterpenes [40, 42, 49] have been identified refer to the higher contribution for the discrimination of the two part samples. It suggested that the differentiation between the inner part and the epidermis may be relative to these chemical components.

**Table 4:** Misclassification table of different parts of *W*. *extensa* using the developed PLS-DA method.

	Members	Correct	Inner part	Epidermis	No class (YPred < 0)
Inner part	30	100%	30	0	0
Epidermis	30	100%	0	30	0
No class	0		0	0	0
Total	60	100%	30	30	0

### Hierarchical Cluster Analysis

**Fig 7:**
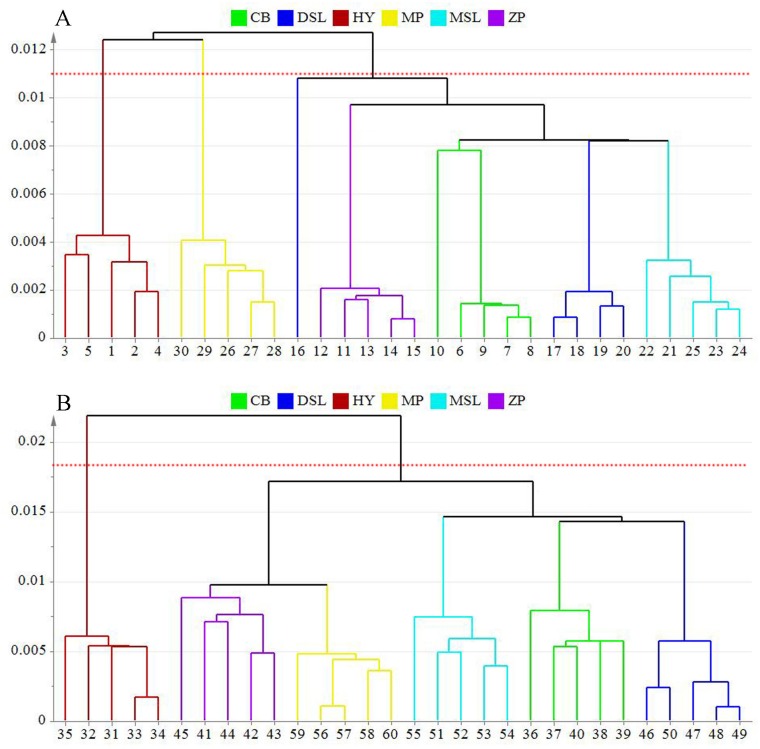
Dendrograms resulting of HCA for the inner part (A) and epidermis (B) *W*. *extensa* samples from different collection sites.

To confirm the previous results concluded by the supervised method of PLS-DA, the unsupervised pattern recognition method HCA was applied on the basis of the optimal PCs obtained by PLS-DA. Single linkage method was used for cluster building. The dendrograms presented in Fig 7A and 7B provide a very simple vision about the degree of similarity among samples. In both cases, samples with the same origins are identified by matching the uniform color. As can be seen in Fig 7A, it clearly shows that all the inner part samples are clustering as their sources except one collected from Dawen Township, Shuangjiang County of Lincang, suggesting a regional dependence of these samples. Overall, three main groups are shown in this dendrogram and samples cluster in the same group means a large similarity on their chemical constituents. Two of these groups represent the samples obtained from Hongta District of Yuxi and Mojiang County of Pu’er, respectively while another one consisted of other samples, which are in agreement with the results established using PLS-DA. Combined with the detailed information of samples, except for geographical origins, the other difference among samples was the cultivation method. From the results of HCA, samples collected from Hongta District in Yuxi which cultivated in soil-less condition are separated from most of the soil-cultured samples. It means that cultivation method may have a few influences on the inner part of *W*. *extensa*.

For the epidermis (Fig 7B), the classification of samples is identical to their collection sites, which corresponds to the results of PLS-DA. On the whole, it also shows the clustering of these samples in two major separated groups. One contains samples originated from Hongta District in Yuxi which cultivated without soils and the other one consists of all the other samples that covered by red or yellow soils during the cultivation, showing the obvious diversity on chemical constituents between the two groups. Considering the information presented in Table 1, it could be inferred from the results that cultivation method may have a great effect on the chemical constituent of epidermis of *W*. *extensa*. In addition, samples which cultivated in soil culture could be further divided into two subfractions. Apparently, the epidermis collected from Mojiang and Zhenyuan Counties of Pu’er join to form the first fraction and the remaining samples reflect interdependent relationships. Interestingly, samples with the geographical origins of Mojiang and Zhenyuan Counties of Pu’er were covered by red soils in the course of the cultivation in the fields while others were covered by yellow soils. It was possible to estimate that the epidermis samples covered by the soils with same color may have similar chemical properties. Collectively, both the cultivation method and geographical origins may have influences on the chemical constituents of cultivated *W*. *extensa*.

Cultivation is one of the effective ways to resolve resource shortage of wild-grown edible mushrooms and the improvement of the cultivation method of the edible mushrooms has gained an unequivocal and considerable attention in many regions [51]. Mushrooms could be farmed under ambient conditions designed to suit their growth. In general, the different methods used in cultivated mushrooms do have effects on the morphology of fruiting bodies, yield percentage, functional, organoleptic and chemical properties as well as the quality of mushrooms [52]. For example, Roy et al. [53] analyzed the *G*. *lucidum* cultivated with the sawdusts of five woods and found the difference in yield among samples. Oyetayo and Ariyo investigated the differences among micro and macronutrient content of *Pleurotus pulmonarius* cultivated on different woody substrates [52]. Similarly, authors Islam et al. [54] compared the *P*. *ostreatus* cultivated under different conditions and found significant differences among the quality of mushrooms. These researches above-mentioned were similar to the results of our study. What’s more, in our paper, although the cultivation methods could affect the chemical constituents of cultivated *W*. *extensa*, the two parts of mushroom samples revealed different degrees based on the influences. There were much greater influences on the epidermis than the inner parts. However, it is difficult to infer the specific causes of this phenomenon.

On the other hand, external environment plays an important role in the variations in the composition and content of chemical compounds in biological samples. Different kinds of environmental factors including elevation, temperature, humidity, topography and soil may have different extents of effects on the qualities of biological samples even though they come from the same species [55]. Based on the results of our study, we found that the chemical properties of epidermis samples covered by the soils with same color appeared to be similar. For the soil-cultured *W*. *extensa*, samples should be covered by soils during the process of cultivation. So, the epidermis was in touch with the soil closely. However, there were obvious differences in the properties of soils with various colors. For example, the red soil was rich in iron and aluminum oxides, and the black soil contained abundant organic matters; some researchers also found that the deeper the color of soils, the richer the amount of some available trace elements such as magnesium, iron, copper, manganese and zinc [56, 57]. In addition, the properties of soils had some influences on the chemical composition of biological samples [58]. Guo et al. [59] reported that the content of inorganic elements in soil had a negative effect on the accumulation of chemical constituents in *Scutellaria baicalensis*. Yang et al. [60] analyzed the cultivated *Paris polyphylla* var. *yunnanensis* and suggested that among a certain range, the contents of steroidal saponin I, II, VII, and H were correlated with that soil organic matter, pH, available K and P. Therefore, for our study, it could be inferred that the chemical constituents in the epidermis of soil-cultured *W*. *extensa* may be affected by the properties of agricultural soils in the cultivation areas. However, more researches are needed to investigate this special relationship. What’s more, when we choose the cultivation regions of *W*. *extensa*, the soil property should be considered necessarily.

## Conclusion

In this paper, cultivated *W*. *extensa* with different collection regions were successfully compared by an integrated method based on FT-IR and UFLC with the aid of multivariate analysis. As expected, obvious differences as well as the similarities in both different parts and origins of the mushroom samples could be easily identified. In a word, the chemical constituents of cultivated *W*. *extensa* could be attributed to the cultivation methods and geographical origins and all the samples showed regional dependence. However, it also demonstrated that the cultivation methods and geographical origins had much greater influences on the epidermis samples than the inner parts. In other words, the inner part displayed better consistency in quality. What’s more, an interesting finding implied that the agricultural soil properties of cultivation regions may be correlated with the chemical constituents of the epidermis of soil-cultured *W*. *extensa*, rather than the inner parts. Overall, these results could provide a comprehensive chemical evidence for the critical complement of current studies on the quality evaluation of cultivated *W*. *extensa*.

## Supporting Information

S1 Fig The edible mushrooms products appeared on the shelves in supermarkets. (JPG)

S2 Fig The edible mushrooms have been cooked in the daily life. (JPG)

S3 Fig Fuling jiabing, a traditional food of Beijing, which also known as Tuckahoe Pie. (JPG)

S4 Fig Samples from Hongta District in Yuxi, which were cultivated via soil-less pattern, growing on the sawn-off roots of old, dead pine trees covered by the plastic films in an indoor environment. (JPG)
