# Supplementary figures and images for: A Comprehensive and Comparative Study of Wolfiporia extensa Cultivation Regions by Fourier Transform Infrared Spectroscopy and Ultra-Fast Liquid Chromatography
Source: PLoS One. 2016 Dec 30;11(12):e0168998. doi: 10.1371/journal.pone.0168998 (PMC5201297; doi:10.1371/journal.pone.0168998)

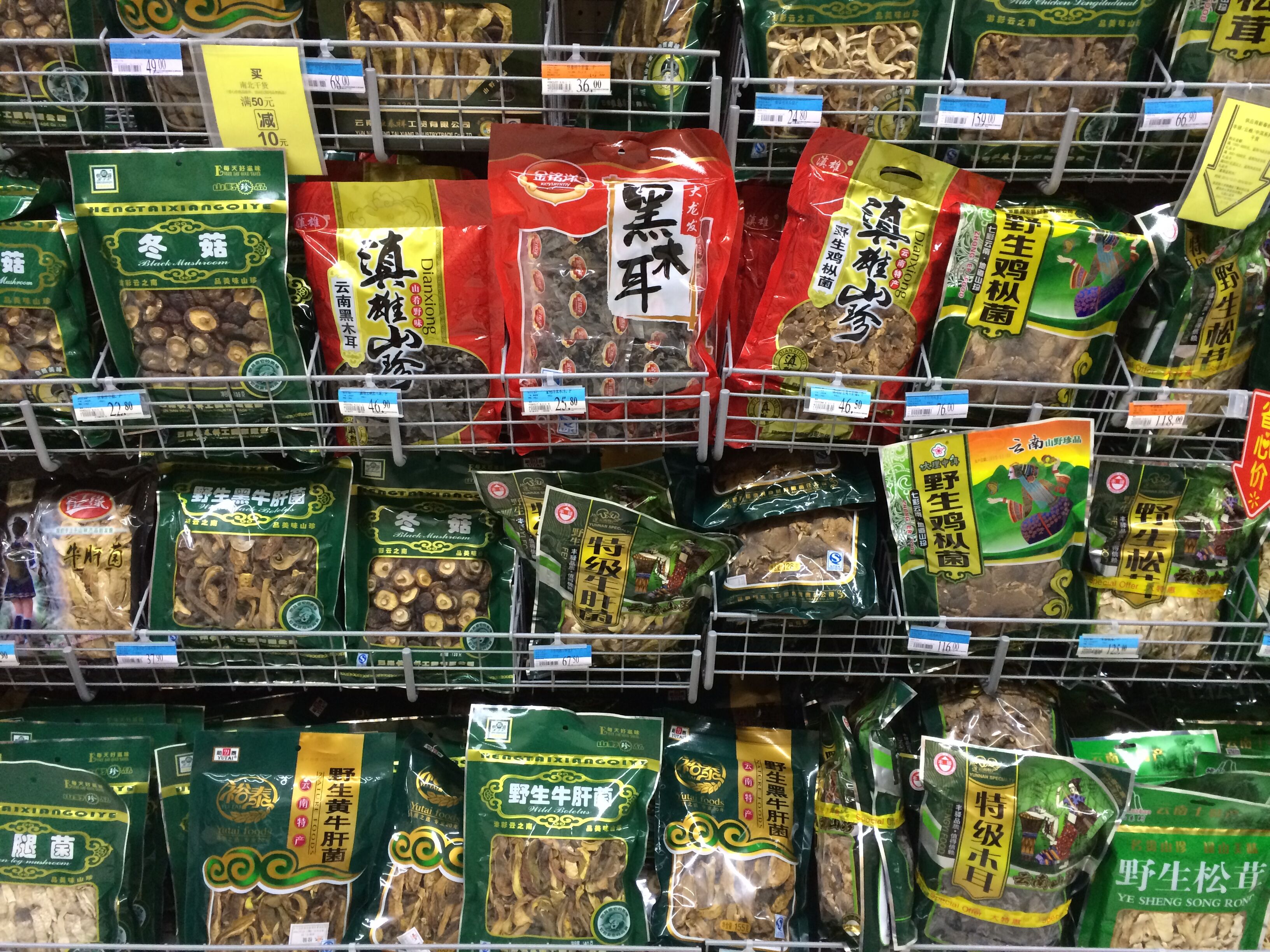

Supplement: S1 Fig — (JPG) [file pone.0168998.s001.jpg]

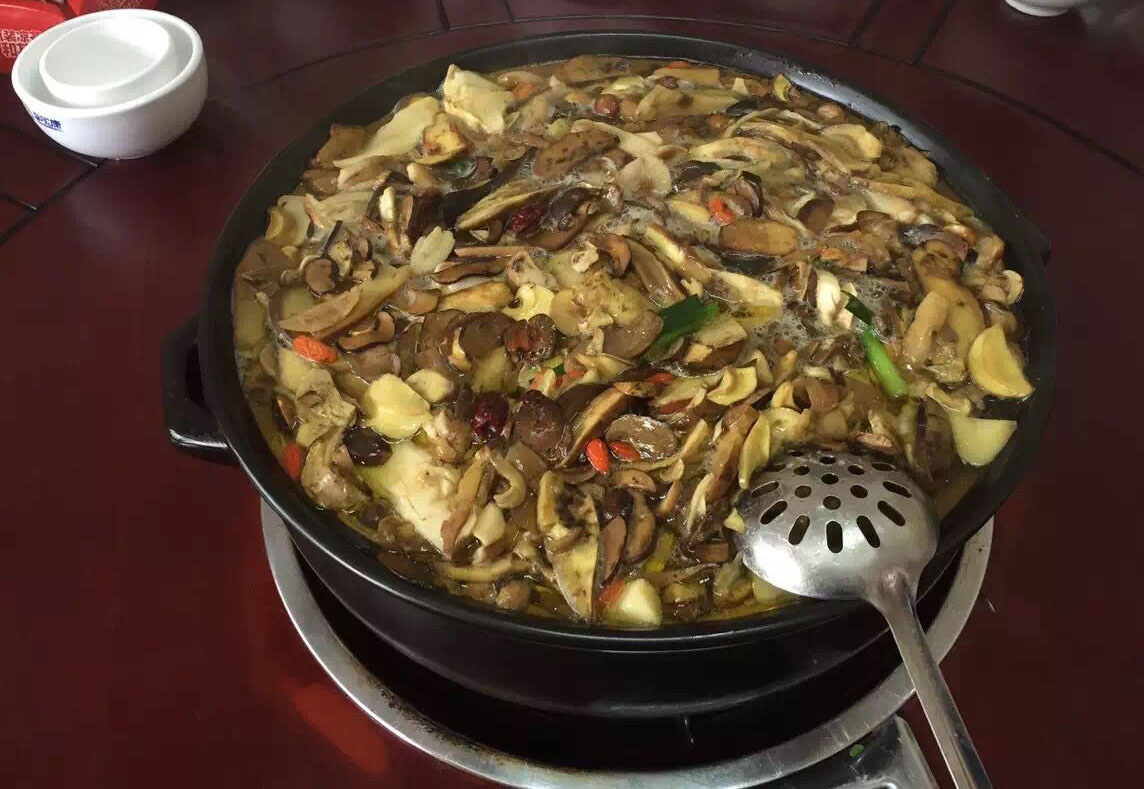

Supplement: S2 Fig — (JPG) [file pone.0168998.s002.jpg]

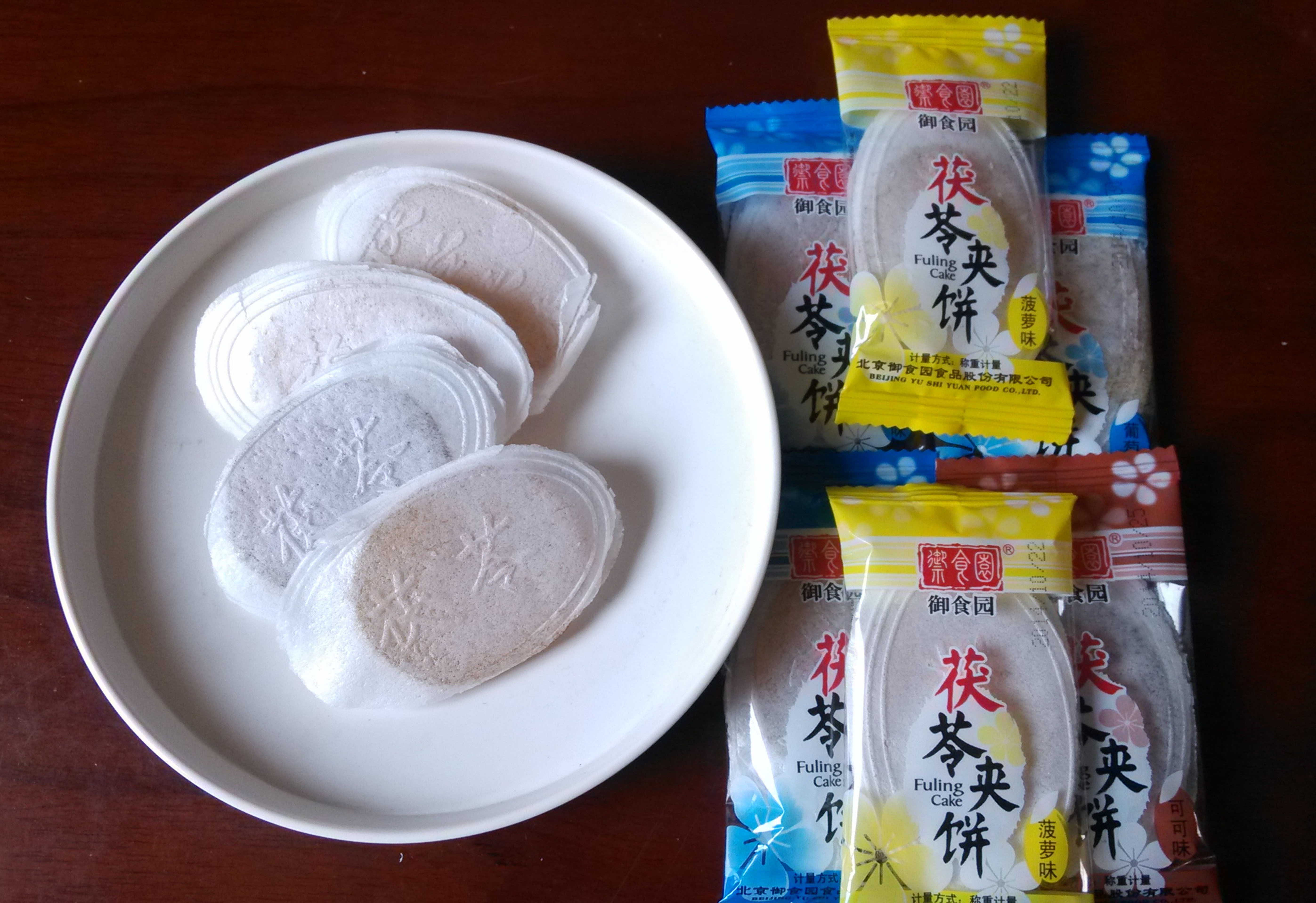

Supplement: S3 Fig — (JPG) [file pone.0168998.s003.jpg]

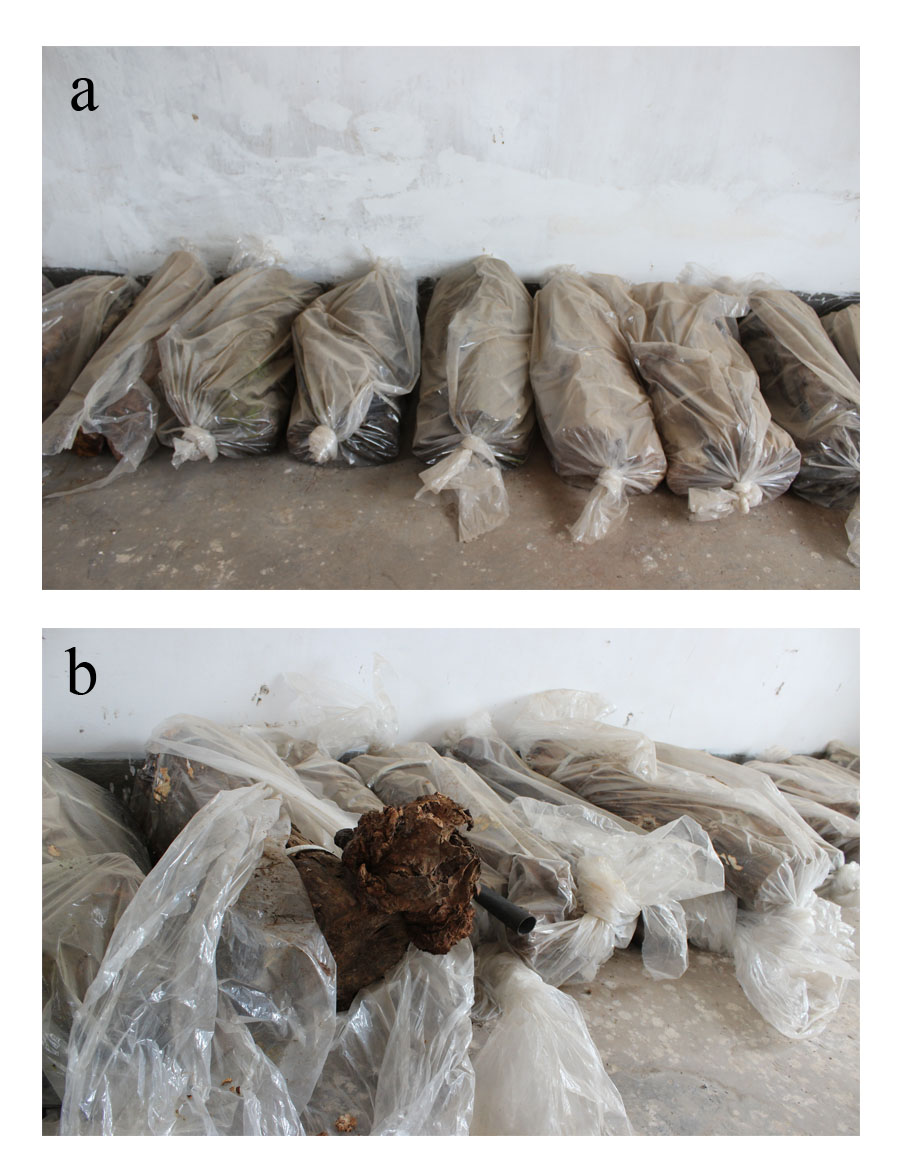

Supplement: S4 Fig — (JPG) [file pone.0168998.s004.jpg]
